# Supplementary material for: CD4 and CD8 Lymphocyte Counts as Surrogate Early Markers for Progression in SARS-CoV-2 Pneumonia: A Prospective Study
Source: Viruses. 2020 Nov 9;12(11):1277. doi: 10.3390/v12111277 (PMC7695272; doi:10.3390/v12111277)
Supplement: Supplementary file 1 [file viruses-12-01277-s001.pdf]

viruses

Supplementary Materials: CD4 and CD8 Lymphocyte Counts as Surrogate Early Markers for Progression in SARS-CoV-2 Pneumonia: A Prospective Study

Supplementary Statistical methods

Summary of clinical data and laboratory parameters and their association with disease evolution were performed using non-parametric methods. Medians, interquartile ranges, and Mann–Whitney tests were used for continuous measures, while frequencies and exact Fisher's test were applied to categorical variables. Spearman correlation coefficients (SCC) and their corresponding 95% confidence intervals (CI) were used to assess association of blood parameters with length of stay at hospital.

Linear regression models were used to assess associations with outcomes when statistical control for confounders was needed (age, gender, and time from symptoms onset). Adjusted group means derived from the models and Partial Correlation Coefficients (PCC) and their corresponding 95% CI were used to display the magnitude of the effects. Results were graphically represented in boxplots, strip charts, or scatter plots as suitable. When needed, Tukey's transformation was applied to the continuous variables in order to fulfill the assumptions of the linear model. Lambda parameters selected for transformations were 0 (i.e., logarithmic transformation: Days of hospitalization, Leucocyte count, Neutrophil count, Lymphocyte count, Ratio N/L, Ferritin, CD3+CD4+ %, CD3+CD4+ count, CD3+CD8+ %, CD3+CD8+ count, CD3+CD4+CD8+ %, CD3+CD4+CD8+ count, CD3+CD4−CD8− %, CD3+CD4−CD8− count, Ratio CD4+/CD8+, CD4+ MFI and CD8+ MFI); 0.5 (i.e., square root: Days of symptoms onset and CRP (mg/dL), T lymphocyte count, B Lymphocyte %, B Lymphocyte count, Natural Killer %, Natural Killer count); −0.5 (i.e., inverse of square root: D-Dimer, LDH); and 2 (i.e., raised to the power of 2: T lymphocyte %). Adjusted means estimated by the models were transformed back to the variable's original scale to enable interpretation of the results.

The predictive value of each blood determination was independently assessed using its Receiver Operating Characteristic (ROC) and the corresponding Area Under the Curve (AUC). To assess the prediction ability of pre-selected combinations of markers, a logistic regression model was fitted to disease evolution in which markers were included as explanatory variables. In addition, blood determinations were simultaneously evaluated and prioritized according to their predictive power in a multivariate setting in an agnostic way. For doing so, we used logistic regression via LASSO penalization of the maximum likelihood [33], as implemented in the R package *glmnet* [34]. The LASSO model is a statistical learning model that performs regularization and variable selection simultaneously and is suitable for scenarios with a high number of variables. LDH was excluded from the later analysis because values were not available for one-third of the patients (10) in this determination. In order to avoid model overfitting, markers combinations were evaluated using a leave-one-out cross-validation process. ROCs were computed on the probabilities predicted by the model for the samples that had been left out in each cross-validation instance. Intervals at 95% confidence were computed for AUCs using bootstrap [35]. The total accuracy, sensitivity, and specificity were computed for an optimal threshold, which is defined as the ROC point closest to the top-left part of the plot (perfect sensitivity and specificity). Of note, the later performance metrics are reported for illustration purposes only, as the threshold selection did not undergo a cross-validation procedure.

Five percent was set as the threshold for statistical significance. All statistical analyses were conducted with R [36].

Table S1. Patients characteristics and blood measurements at time of admission by gender groups.

	**All (*n* = 30)**	**Male 20 (66.7%)**	**Female 10 (33.3%)**	***p*-value**
Age	60.615 [56.099, 63.266]	56.821 [51.737, 62.294]	62.901 [59.348, 75.562]	0.0713
Days of symptoms onset	7.000 [6.000, 10.000]	9.000 [6.000, 10.000]	5.500 [3.000, 11.000]	0.1511
Days of hospitalization	8.000 [5.000, 14.000]	8.500 [4.000, 14.000]	6.500 [4.000, 22.000]	0.5079
HT	6 (20.0%)	3 (50.0%)	3 (50.0%)	0.3432
DM	1 (3.3%)	0 (0.0%)	1 (100.0%)	0.1322
DLP	5 (16.7%)	3 (60.0%)	2 (40.0%)	0.7320
OBESITY	1 (3.3%)	0 (0.0%)	1 (100.0%)	0.1322
Leucocyte count (cells × 10^9/L)	6310.000 [5310.000, 8860.000]	6680.000 [5680.000, 9050.000]	5555.000 [3680.000, 13310.000]	0.2263
Neutrophil count (cells × 10^9/L)	4440.000 [3920.000, 6650.000]	4760.000 [4200.000, 7030.000]	3595.000 [2860.000, 11740.000]	0.3789
Lymphocyte count (cells × 10^9/L)	1215.000 [1040.000, 1310.000]	1295.000 [1070.000, 1710.000]	965.000 [810.000, 1260.000]	0.0221
Ratio N/L	4.264 [3.049, 5.076]	4.422 [2.741, 5.185]	3.947 [1.651, 11.946]	0.7249
Ferritin (ng/mL)	711.700 [382.600, 1136.200]	969.100 [475.500, 1374.600]	365.050 [109.300, 1722.500]	0.0585
CRP (mg/dL)	8.800 [5.070, 11.250]	8.800 [5.320, 11.440]	7.130 [3.260, 15.670]	0.7581
D-Dimer (mg/mL)	691.000 [443.000, 860.000]	458.500 [335.000, 860.000]	761.500 [679.000, 1213.000]	0.0430
LDH (U/L)	282.500 [244.000, 365.000]	354.000 [244.000, 446.000]	258.000 [205.000, 401.000]	0.1105
T lymphocyte %	68.660 [60.140, 74.040]	65.415 [54.930, 74.880]	72.405 [60.140, 81.790]	0.1726
T lymphocyte count	713.500 [497.000, 823.000]	685.000 [451.000, 823.000]	740.500 [413.000, 1119.000]	0.7749
CD3+CD4+ %	41.975 [38.050, 48.820]	40.570 [32.670, 48.540]	45.560 [33.820, 56.100]	0.5093
CD3+CD4+ count	467.000 [303.000, 574.000]	451.000 [278.000, 747.000]	516.000 [221.000, 767.000]	0.9299
CD3+CD8+ %	18.835 [15.530, 24.740]	18.240 [14.880, 24.740]	21.670 [10.040, 28.460]	0.7581
CD3+CD8+ count	245.000 [171.000, 319.000]	258.000 [171.000, 320.000]	225.000 [71.000, 586.000]	0.6759
CD3+CD4+CD8+ %	1.020 [0.770, 1.830]	1.020 [0.760, 1.920]	1.270 [0.760, 2.580]	0.5672
CD3+CD4+CD8+ count	12.500 [8.000, 21.000]	14.000 [8.000, 25.000]	12.000 [6.000, 26.000]	0.7916
CD3+CD4−CD8− %	1.395 [0.960, 1.940]	1.395 [0.800, 1.960]	1.520 [0.600, 2.470]	0.9124
CD3+CD4−CD8− count	18.000 [12.000, 23.000]	19.500 [10.000, 27.000]	15.500 [6.000, 23.000]	0.7410
B Lymphocyte %	10.070 [8.510, 12.130]	9.950 [6.870, 12.890]	11.925 [3.500, 17.310]	0.7249
B Lymphocyte count	111.500 [78.000, 162.000]	111.500 [69.000, 201.000]	114.000 [38.000, 195.000]	0.5975
Natural Killer %	16.625 [13.950, 21.670]	19.810 [14.250, 26.990]	14.675 [10.000, 18.400]	0.1347

Natural Killer count	196.000 [154.000, 253.000]	231.000 [154.000, 307.000]	159.000 [61.000, 239.000]	0.1082
Ratio CD4+/CD8+	1.905 [1.580, 3.120]	2.025 [1.490, 3.120]	1.760 [1.320, 5.260]	0.8259
CD4+ MFI	24860.500 [22770.000, 26259.000]	24860.500 [22132.000, 26259.000]	24867.000 [20911.000, 28342.000]	0.8088
CD8+ MFI	25855.500 [23819.000, 27476.000]	25979.000 [24450.000, 29730.000]	24578.000 [21666.000, 30016.000]	0.3011

Ratio N/L: ratio neutrophil to lymphocyte, CRP: C-reactive protein, LDH: lactate dehydrogenase, MFI: median fluorescence intensity. Group medians and percentiles 25 and 75 (continuous variables) or absolute and relative frequencies (categorical variables) are showed. P-values are derived from a Mann–Whitney test (continuous variables) or an exact Fisher's test (binary variables).

Table S2. Lymphocyte subsets percentages and their association with COVID-19 evolution before (Univariate) and after (Adjusted) statistical control for age, gender, and time from symptoms onset.

	**All**	**Univariate**			**Adjusted**		
	**Median [5–75 Pcts]**	**Non-critical Median [25–75 Pcts]**	**Critical Median [25–75 Pcts]**	***p* value**	**Non-critical Adj. Mean [95% CI]**	**Critical Adj. Mean [95%CI]**	***p* value**
T lymphocyte %	68.660 [60.140, 74.040]	70.820 [60.140, 76.360]	65.610 [54.930, 74.880]	0.4898	69.031 [62.707, 74.822]	66.554 [58.948, 73.376]	0.5927
CD3+CD4+ %	41.975 [38.050, 48.820]	48.820 [40.430, 57.210]	38.020 [29.110, 42.240]	0.00363	48.000 [42.376, 54.371]	34.998 [30.341, 40.370]	0.00223
CD3+CD8+ %	18.835 [15.530, 24.740]	17.260 [14.850, 24.730]	24.040 [14.460, 37.010]	0.0687	17.234 [13.869, 21.416]	22.919 [17.869, 29.398]	0.0899
CD3+CD4+CD8+ %	1.020 [0.770, 1.830]	1.030 [0.770, 1.830]	1.010 [0.480, 2.570]	0.7855	1.224 [0.850, 1.762]	1.183 [0.780, 1.796]	0.9020
CD3+CD4-CD8- %	1.395 [0.960, 1.940]	1.650 [1.270, 2.100]	1.040 [0.760, 1.940]	0.2497	1.561 [1.080, 2.255]	1.109 [0.727, 1.691]	0.2233
B Lymphocyte %	10.070 [8.510, 12.130]	11.990 [6.870, 12.890]	9.840 [6.330, 17.310]	0.6009	10.098 [7.183, 13.508]	10.657 [7.260, 14.705]	0.8166
Natural Killer %	16.625 [13.950, 21.670]	15.580 [10.990, 20.860]	16.840 [13.770, 32.460]	0.3254	16.142 [12.132, 20.724]	20.013 [14.907, 25.869]	0.2626

Group medians and percentiles 25 and 75 (Pcts) are displayed for description purposes. Adjusted means (Adj mean) and their 95% confidence intervals (CI) are showed when controlling by confounders. P-values are derived from Mann–Whitney test (Univariate) or from the F-test of a linear model (Adjusted).

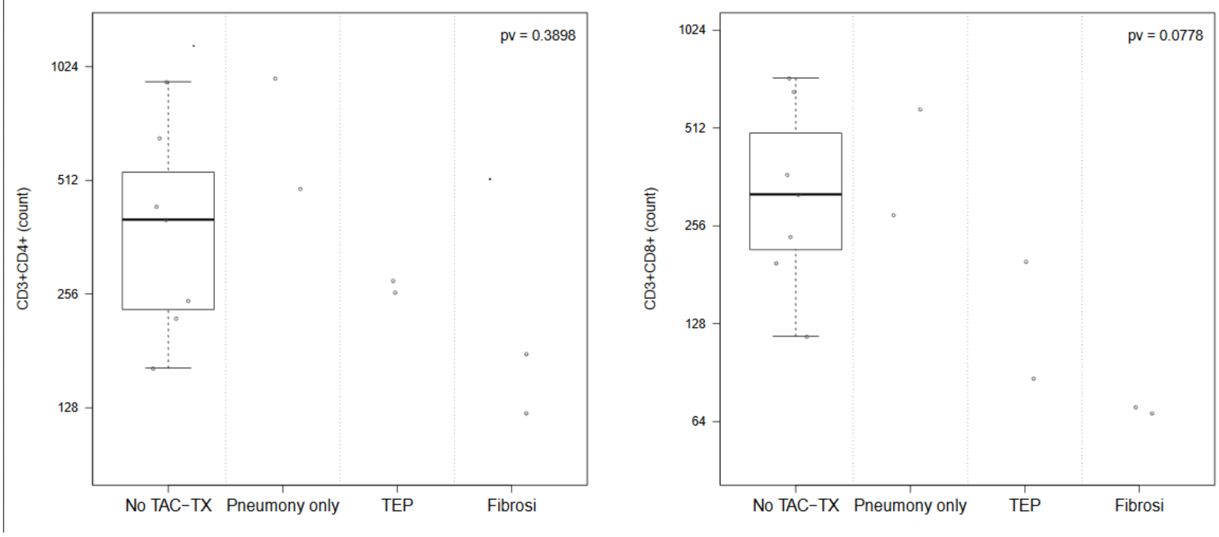

Figure S1. Boxplots and strip charts showing for CD3+CD4+ T cell count (left) and CD3+CD8+ T cell count (right) by diagnosis from Thorax computerized axial tomography in COVID-19 patients that reached a critical clinical condition of the disease and performed following clinical practice criteria.

Several authors have demonstrated that the neutralization of CD4 and/or CD8 T cells in mice after injecting anti-CD3, anti-CD4, or anti-CD8 antibodies reduced the intensity of lung fibrosis. Recent studies strongly suggest that particular subpopulations of CD4 T lymphocytes accumulate in lung fibrotic disorders and play a pivotal role in fibrogenesis [37]. In contrast to these data, we observed that the lowest CD3+CD4+ and CD3+CD8+ T cells counts were detected in the two patients with pulmonary fibrosis. Although requiring further confirmation, these results are in agreement with previous data published on the ADRS associated to CMV infection [38]. In addition, a decrease in peripheral lymphocyte count may be related to the cell mobilization and concentration to specific affected areas such as lungs, which is induced by the presence of the pathogen. A longer monitoring of these patients appears to be mandatory to evaluate this outcome accurately.

Table S3. Association of patient characteristics and blood determinations with length of stay at hospital in COVID-19 patients with non-critical and aggravated (critical) evolution of the disease.

	**Non−critical**		**Critical**	
	**SCC [95%CI]**	***p* val**	**SCC [95%CI]**	***p* val**
Age	0.492 [−0.064, 0.829]	0.0449	0.302 [−0.437, 0.772]	0.3405
Days of symptoms onset	−0.118 [−0.556, 0.337]	0.6518	−0.131 [−0.796, 0.467]	0.6839
Leucocyte count (cells × 10^9/L)	−0.199 [−0.623, 0.312]	0.4432	0.158 [−0.534, 0.757]	0.6241
Neutrophil count (cells × 10^9/L)	−0.154 [−0.606, 0.322]	0.5540	−0.081 [−0.716, 0.618]	0.8031
Lymphocyte count (cells × 10^9/L)	−0.589 [−0.830, −0.184]	0.0128	0.316 [−0.385, 0.826]	0.3165
Ratio N/L	0.146 [−0.354, 0.578]	0.5768	−0.126 [−0.785, 0.673]	0.6957
Ferritin (ng/mL)	0.144 [−0.457, 0.629]	0.5801	0.112 [−0.518, 0.718]	0.7283
CRP (mg/dL)	0.238 [−0.338, 0.708]	0.3579	0.004 [−0.560, 0.603]	0.9914
DDimer (mg/mL)	−0.273 [−0.668, 0.237]	0.2895	0.488 [−0.091, 0.773]	0.1077

LDH (U/L)	0.188<br>[−0.506, 0.859]	0.5806	0.012<br>[−0.981, 0.646]	0.9775
T lymphocyte %	−0.085<br>[−0.675, 0.488]	0.7466	−0.256<br>[−0.771, 0.377]	0.4216
T lymphocyte count	−0.016<br>[−0.523, 0.470]	0.9508	−0.316<br>[−0.775, 0.431]	0.3173
CD3+CD4+ %	−0.179<br>[−0.623, 0.371]	0.4910	0.312<br>[−0.267, 0.788]	0.3230
CD3+CD4+ count	−0.218<br>[−0.655, 0.288]	0.4007	−0.137<br>[−0.708, 0.704]	0.6715
CD3+CD8+ %	−0.219<br>[−0.707, 0.291]	0.3979	−0.407<br>[−0.871, 0.315]	0.1891
CD3+CD8+ count	−0.325<br>[−0.703, 0.127]	0.2027	−0.439<br>[−0.903, 0.311]	0.1538
CD3+CD4+CD8+ %	0.090<br>[−0.457, 0.588]	0.7320	−0.018<br>[−0.550, 0.573]	0.9568
CD3+CD4+CD8+ count	−0.082<br>[−0.589, 0.439]	0.7531	−0.261<br>[−0.836, 0.418]	0.4134
CD3+CD4−CD8− %	0.155<br>[−0.323, 0.585]	0.5537	−0.014<br>[−0.614, 0.527]	0.9655
CD3+CD4−CD8− count	−0.020<br>[−0.498, 0.456]	0.9395	−0.350<br>[−0.846, 0.282]	0.2650
B Lymphocyte %	−0.041<br>[−0.663, 0.544]	0.8755	0.028<br>[−0.637, 0.585]	0.9310
B Lymphocyte count	−0.186<br>[−0.679, 0.429]	0.4758	−0.214<br>[−0.724, 0.437]	0.5041
Natural Killer %	0.286<br>[−0.262, 0.733]	0.2650	−0.042<br>[−0.694, 0.674]	0.8966
Natural Killer count	0.125<br>[−0.425, 0.684]	0.6320	−0.172<br>[−0.752, 0.572]	0.5931
CD4+/CD8+ Ratio	−0.012<br>[−0.461, 0.563]	0.9640	0.351<br>[−0.483, 0.777]	0.2634
CD4+ MFI	−0.290<br>[−0.666, 0.215]	0.2585	−0.295<br>[−0.718, 0.307]	0.3524
CD8+ MFI	−0.212<br>[−0.579, 0.228]	0.4146	−0.396<br>[−0.857, 0.242]	0.2019

Ratio N/L: ratio neutrophils to lymphocytes, CRP: C-reactive protein, LDH: lactate dehydrogenase, MFI: median fluorescence intensity. Spearman Correlation Coefficients (SCC) and their 95% corresponding confidence intervals (CI) and *p*-values are showed.

Table S4. Association of patient characteristics and blood determinations with length of stay at hospital in COVID-19 patients with non-critical and aggravated (Critical) evolution of the disease, after statistical control by age, gender, and time from symptoms onset.

	**Non−critical**		**Critical**	
	**Medians/Spearm.Cor r [95%CI]**	**Kruskal/Spearman pval**	**Medians/Spearm.Cor r [95%CI]**	**Kruskal/Spearman pval**
Age	0.548 [0.049, 0.828]	0.0346	0.502 [−0.187, 0.860]	0.1396
Days of symptoms onset	−0.210 [−0.652, 0.339]	0.4532	0.436 [−0.267, 0.836]	0.2079
Leucocyte count (cells × 10^9/L)	−0.433 [−0.784, 0.126]	0.1217	0.186 [−0.545, 0.757]	0.6314
Neutrophil count (cells × 10^9/L)	−0.408 [−0.772, 0.156]	0.1475	0.069 [−0.624, 0.701]	0.8595
Lymphocyte count (cells × 10^9/L)	−0.459 [−0.796, 0.095]	0.0991	0.247 [−0.499, 0.783]	0.5222
Ratio N/L	−0.185 [−0.652, 0.383]	0.5262	−0.083 [−0.708, 0.615]	0.8326
Ferritin (ng/mL)	0.463 [−0.089, 0.798]	0.0953	0.045 [−0.638, 0.688]	0.9092
CRP (mg/dL)	0.420 [−0.142, 0.777]	0.1349	−0.061 [−0.697, 0.629]	0.8768
D-Dimer (mg/mL)	−0.378 [−0.757, 0.191]	0.1825	0.039 [−0.642, 0.686]	0.9200
LDH (U/L)	−0.113 [−0.757, 0.643]	0.7902	−0.772 [−0.984, 0.345]	0.1258
T lymphocyte %	−0.140 [−0.624, 0.422]	0.6331	−0.485 [−0.869, 0.264]	0.1855
T lymphocyte count	−0.057 [−0.570, 0.489]	0.8474	−0.601 [−0.904, 0.105]	0.0869
CD3+CD4+ %	−0.222 [−0.673, 0.350]	0.4453	−0.008 [−0.668, 0.660]	0.9844
CD3+CD4+ count	−0.238 [−0.683, 0.334]	0.4117	−0.548 [−0.889, 0.182]	0.1262
CD3+CD8+ %	−0.130 [−0.618, 0.430]	0.6568	−0.463 [−0.862, 0.291]	0.2097
CD3+CD8+ count	−0.237 [−0.682, 0.335]	0.4137	−0.700 [−0.931, −0.066]	0.0359
CD3+CD4+CD8+ %	−0.050 [−0.566, 0.493]	0.8642	0.203 [−0.533, 0.764]	0.6003
CD3+CD4+CD8+ count	−0.168 [−0.641, 0.398]	0.5662	−0.175 [−0.752, 0.553]	0.6525
CD3+CD4−CD8− %	−0.231 [−0.678, 0.342]	0.4278	0.469 [−0.284, 0.864]	0.2031
CD3+CD4−CD8− count	−0.265 [−0.697, 0.309]	0.3602	−0.134 [−0.733, 0.582]	0.7320
B lymphocyte %	−0.158 [−0.635, 0.407]	0.5907	0.195 [−0.539, 0.761]	0.6148
B lymphocyte count	−0.196 [−0.658, 0.374]	0.5027	−0.250 [−0.784, 0.497]	0.5172

Natural killer %	0.373 [−0.197, 0.754]	0.1893	0.201 [−0.535, 0.763]	0.6049
Natural killer count	0.222 [−0.349, 0.674]	0.4447	−0.500 [−0.874, 0.245]	0.1701
CD4+/CD8+ Ratio	−0.012 [−0.539, 0.522]	0.9664	0.347 [−0.412, 0.822]	0.3599
CD4+ MFI	−0.869 [−0.958, −0.627]	5.56e−05	−0.175 [−0.752, 0.553]	0.6518
CD8+ MFI	−0.227 [−0.676, 0.346]	0.4360	−0.462 [−0.862, 0.292]	0.2110

Ratio N/L: ratio neutrophils to lymphocytes, CRP: C-reactive protein, LDH: lactate dehydrogenase, MFI: median fluorescence intensity. Partial Correlation Coefficients (PCC) and their 95% corresponding confidence intervals (CI) are showed. P-values are derived from the F-test of the corresponding linear model.

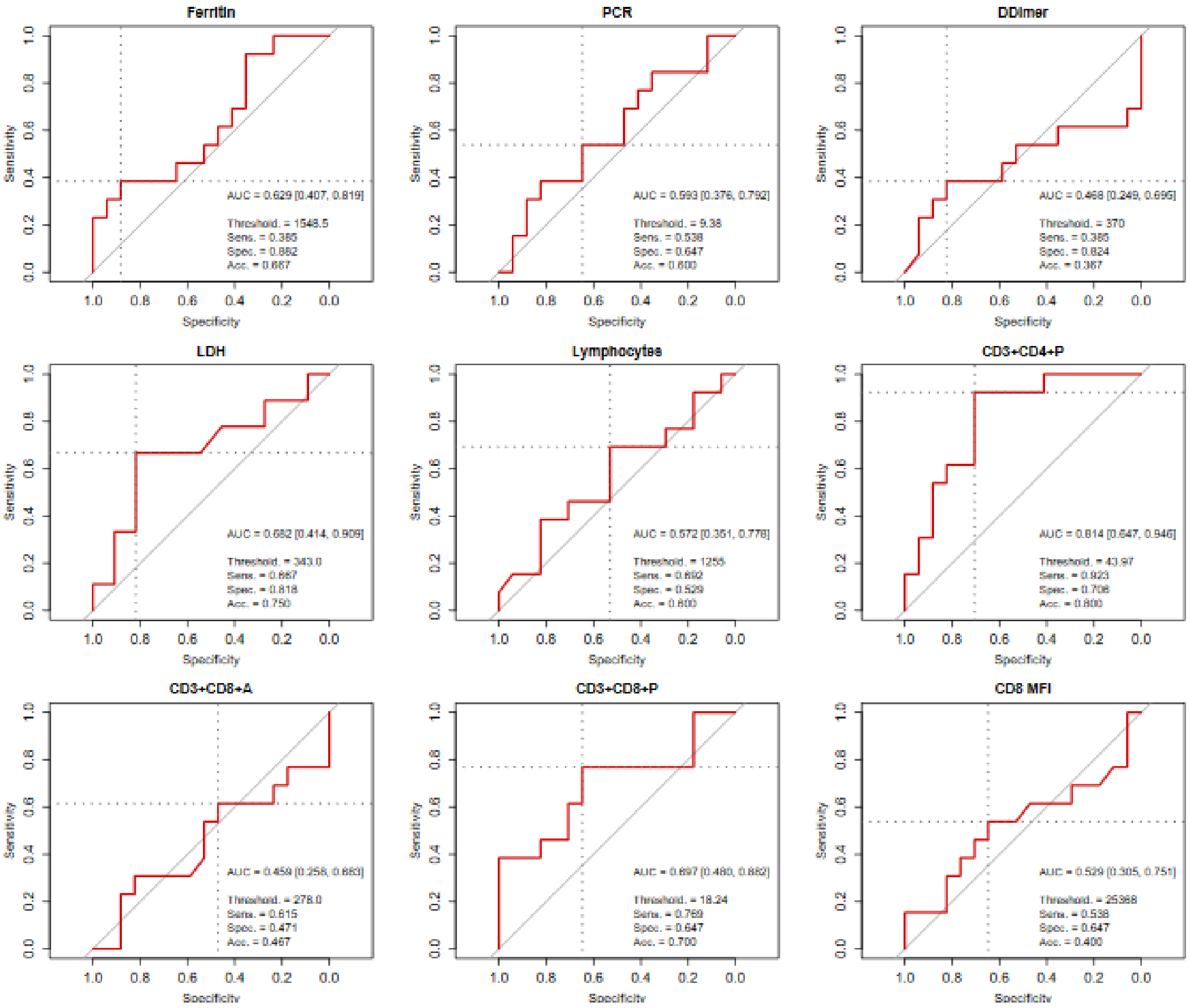

Figure S2. Receiver Operating Characteristics (ROC) and their corresponding Area Under the Curve (AUC) for the prediction of COVID-19 clinical evolution in blood determinations. AUC intervals at 95% confidence were computed using bootstrap. Total accuracy, sensitivity, and specificity are displayed for the optimal threshold, which is defined as the ROC point closest to the top-left part of the plot (perfect sensitivity and specificity).

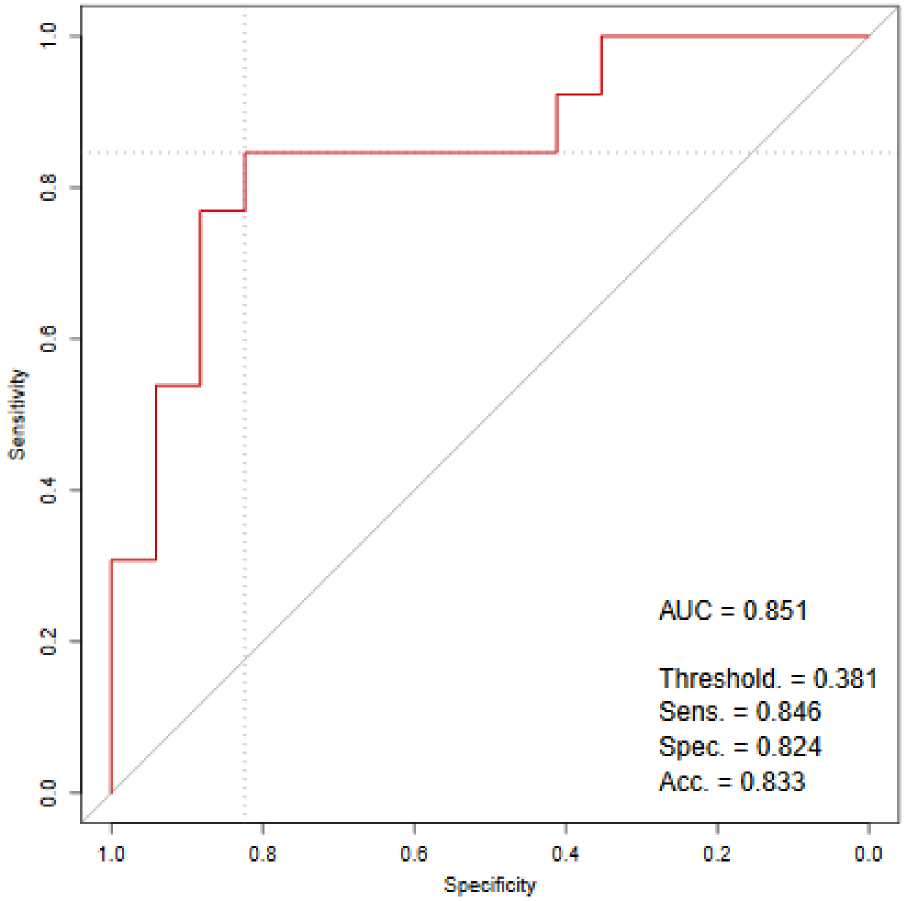

Figure S3. Receiver Operating Characteristics (ROC) and their corresponding Area Under the Curve (AUC) for the prediction of COVID-19 clinical evolution using CD4 MFI and CD3+CD4+ T cells simultaneously. ROC curve was computed on the probabilities derived from a logistic regression model fitted to disease evolution (Non-critical vs. Critical) that included CD4 MFI and CD3+CD4+ T cells as explanatory variables. Probabilities were estimated under a leave-one-out cross-validation procedure to avoid model over-fitting. AUC intervals at 95% confidence were computed using bootstrap. Total accuracy, sensitivity, and specificity are displayed for the optimal threshold, which is defined as the ROC point closest to the top-left part of the plot (perfect sensitivity and specificity).

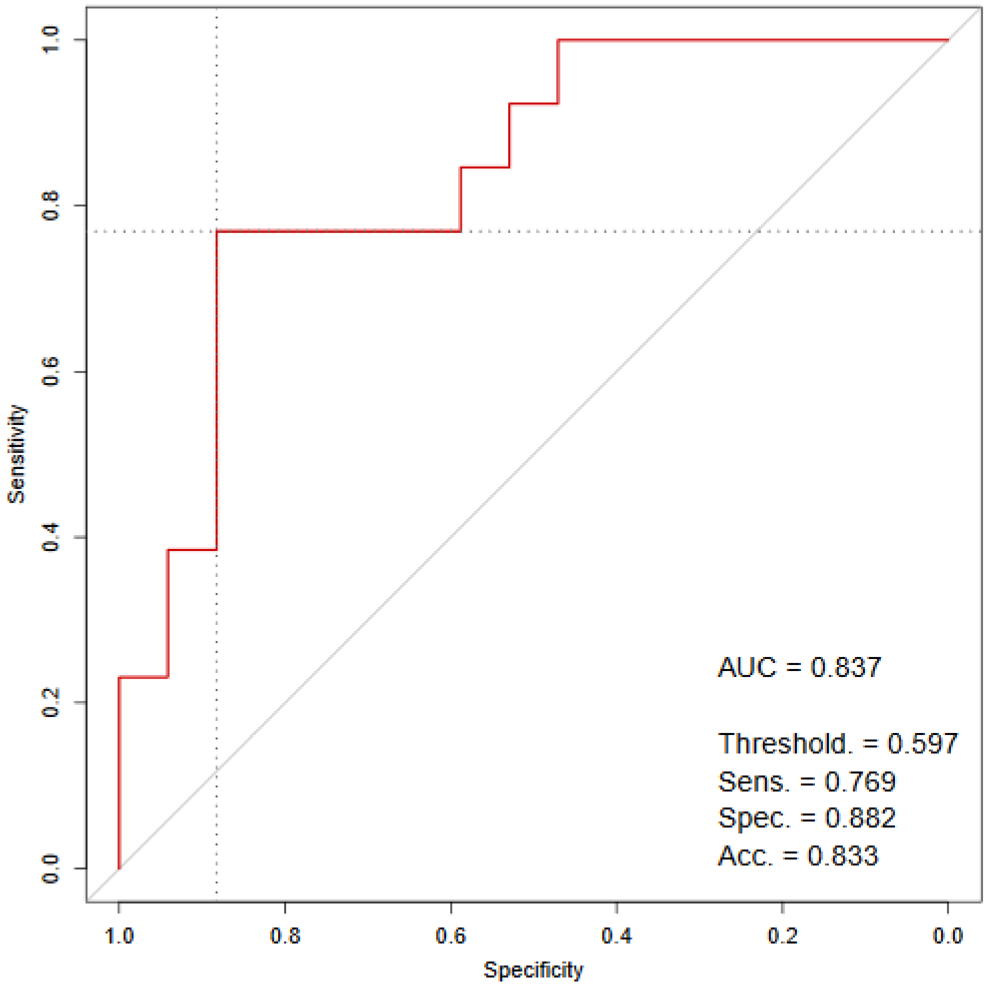

Figure S4. Receiver Operating Characteristics (ROC) and their corresponding Area Under the Curve (AUC) for the prediction of COVID-19 clinical evolution using CD4 MFI and CD3+CD4+ T cell and CD4+/CD8+ ratio simultaneously. ROC curve was computed on the probabilities derived from a logistic regression model fitted to disease evolution (Non-critical vs. Critical) that included CD4 MFI, CD3+CD4+, and CD4+/CD8+ ratio as explanatory variables. Probabilities were estimated under a leave-one-out cross-validation procedure to avoid model over-fitting. AUC intervals at 95% confidence were computed using bootstrap. Total accuracy, sensitivity, and specificity are displayed for the optimal threshold, which is defined as the ROC point closest to the top-left part of the plot (perfect sensitivity and specificity).

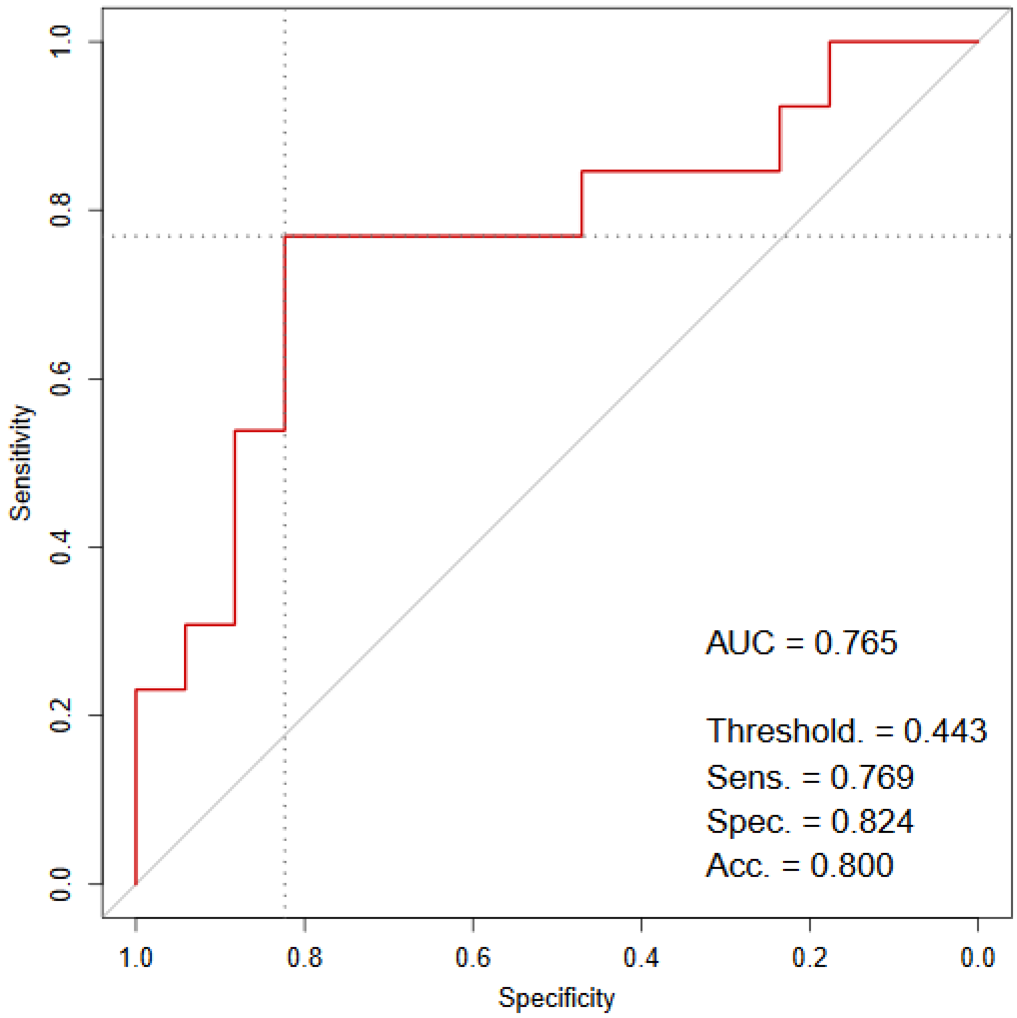

Figure S5. Receiver Operating Characteristics (ROC) and their corresponding Area Under the Curve (AUC) for the prediction of COVID-19 clinical evolution using all blood determinations simultaneously. ROC curve was computed on the probabilities derived from a LASSO logistic regression model fitted to disease evolution (Non-critical vs. Critical) that included age and all blood determinations, with the exception of LDH due to its high number of missing values (10). Probabilities were estimated under a leave-one-out cross-validation procedure to avoid model over-fitting. AUC intervals at 95% confidence were computed using bootstrap. Total accuracy, sensitivity and specificity are displayed for the optimal threshold, defined as the ROC point closest to the top-left part of the plot (perfect sensitivity and specificity).

Table S5. Frequency of blood determinations selection by the LASSO logistic regression model across the 30 instances of the leave-one-out cross-validation procedure.

Measurement	N	%
CD3+CD4+ T cell transf. (alfa = 0, lambda = 0)	30	100.0%
CD3+CD4+CD8+ T cell transf. (alfa = 0, lambda = 0)	2	6.7%
CD4_MFI transf. (alfa = 0, lambda = 0)	30	100.0%
DD1 transf. (alfa = 0, lambda = -0.5)	1	3.3%
CD4+/CD8+ ratio transf. (alfa = 0, lambda = 0)	30	100.0%
